# Supplementary material for: End Sequence Analysis Toolkit (ESAT) expands the extractable information from single-cell RNA-seq data
Source: Genome Res. 2016 Oct;26(10):1397–410. doi: 10.1101/gr.207902.116 (PMC5052061; doi:10.1101/gr.207902.116)
Supplement: Supplemental Material [file supp_gr.207902.116_Supp_Materials.tar.gz › ESAT-esat_v0.1/doc/html/classumms_1_1core_1_1alignment_1_1_alignment_pair.html]

ESAT: umms.core.alignment.AlignmentPair Class Reference


ESAT


- Main Page
- Related Pages
- Packages
- Classes
- Files

- Class List
- Class Index
- Class Hierarchy
- Class Members

All Classes Namespaces Files Functions Variables Enumerator Pages

- umms
- core
- alignment
- AlignmentPair

Public Member Functions |
List of all members

umms.core.alignment.AlignmentPair Class Reference

Inheritance diagram for umms.core.alignment.AlignmentPair:

[legend]

Collaboration diagram for umms.core.alignment.AlignmentPair:

[legend]

|  |  |
| --- | --- |
| Public Member Functions | |
| void | add (SAMRecord record) |
|  | |
| boolean | isComplete () |
|  | |
| Collection< SAMRecord > | makePairs () |
|  | |
| boolean | hasEntries () |
|  | |
| boolean | valuesAreEmpty () |
|  | |

## Detailed Description

Represents a paired alignment in all detail, with possbily multiple mappings through the genome. An alignment pair is meant to be built iteratively as mappings of an insert are found. Once all pieces are assembled, it can do useful things.

Author
:   mgarber

## Member Function Documentation

|  |  |  |  |  |  |
| --- | --- | --- | --- | --- | --- |
| void umms.core.alignment.AlignmentPair.add | ( | SAMRecord | *record* | ) |  |

|  |  |  |  |  |
| --- | --- | --- | --- | --- |
| boolean umms.core.alignment.AlignmentPair.hasEntries | ( |  | ) |  |

Here is the caller graph for this function:

|  |  |  |  |  |
| --- | --- | --- | --- | --- |
| boolean umms.core.alignment.AlignmentPair.isComplete | ( |  | ) |  |

|  |  |  |  |  |
| --- | --- | --- | --- | --- |
| Collection<SAMRecord> umms.core.alignment.AlignmentPair.makePairs | ( |  | ) |  |

|  |  |  |  |  |
| --- | --- | --- | --- | --- |
| boolean umms.core.alignment.AlignmentPair.valuesAreEmpty | ( |  | ) |  |

---

The documentation for this class was generated from the following file:

- java/umms/core/alignment/AlignmentPair.java


---

Generated on Fri Aug 15 2014 15:11:07 for ESAT by  

 1.8.7
